# Survival economics and criminalization among women who use drugs during COVID-19 and de facto drug decriminalization in Baltimore, Maryland

**DOI:** 10.1016/j.dadr.2026.100484

**Published:** 2026-10-12

**Authors:** Natalie Flath, Tali Ziv, Catherine Tomko, Saba Rouhani, Laura Nicole Sisson, Susan G. Sherman

**Affiliations:** a Department of Medicine Global Public Health, University of California San Diego, San Diego, USA; b Department of Anthropology, Texas State University, Rochester, NY, USA; c Department of Health, Behavior, & Society, Johns Hopkins Bloomberg School of Public Health, Baltimore, MD, USA; d Department of Epidemiology, New York University School of Global Public Health, New York, NY, USA.

**Keywords:** Drug policy, Drug decriminalization, Social determinants of health, Survival economics, Women who use drugs

## Abstract

**Background:**

Women who use drugs (WWUD) often experience social and economic marginalization. Many rely on survival economies that span formal, informal, and illicit income-generating activities, which may increase exposure to the criminal legal system. This study identified exploratory patterns of survival economic strategies among WWUD, then examined associations between income activities and criminal legal exposures during Baltimore, Maryland’s COVID-19 emergency period and *de facto* drug decriminalization.

**Methods:**

Data come from the baseline assessment a WWUD cohort (*n* = 227) in Baltimore, recruited from August 2021 to December 2022. Guided by a sociological framework of income sources among low-income women, latent class analysis was used to identify survival economics across state support, instrumental support (money from family, friends, partners), informal labor, and illicit work (selling drugs, sex work). We then assessed associations between two common income sources (illicit and state support) and criminal legal exposures using Poisson regression with robust variance.

**Results:**

Three distinct survival economic strategies emerged: mixed and diverse income (57% of sample), primarily state supported (27%), and primarily illicit (15%). Arrest prevalence did not differ across income activities. However, WWUD reporting illicit income compared to not were significantly more likely to report police encounters (adjusted prevalence ratios (aPR) 1.61 95% CI 1.39, 2.01), including abusive ones, although borderline significant (aPR 1.33 95% CI 1.02,1.74).

**Conclusions:**

WWUD engaged in diverse survival economic strategies and illicit income generation was associated with police contact. Although these findings are exploratory, they highlight the importance of clarifying how criminalized survival economies are policed.

## Highlights

• Latent class analysis identifies distinct survival economy strategies among women who use drugs.
• Strategies span overlapping formal, informal, and illicit income generation.
• Arrests did not vary significantly across women’s income generation strategies.
• Illicit income generation was associated with more police interactions, including abusive ones.
• Policing and survival strategies remain intertwined despite *de facto* decriminalization.

## 1 Introduction

Punitive drug enforcement, or the criminalization of drugs, leads to a wide range of social and health harms. The criminalization of drugs is linked to higher rates of infectious disease transmission (Baker et al., 2020), overdose (Mital et al., 2020), and barriers to critical social dimensions of health. These include employment, housing, and the right to vote (Cohen et al., 2022). Together, this body of research provides the foundation for the growing implementation of decriminalization policies that remove criminal penalties for the possession and use of illicit drugs in major cities across North America (Drug Policy Alliance, 2019).

Despite the promise, emerging evidence shows that lifting criminal penalties for the possession and use of drugs may not prevent the ongoing criminalization of other illegally deemed activities that shape daily survival. Studies have shown how post-decriminalization, criminal charges may net widen and shift to adjacent categories such as traffic-related stops, crimes of poverty (e.g., panhandling) and drug trafficking, continuing to expose people who use drugs to other harms of criminalization (Kozlowski et al., 2019, Levy, 2018, Michaud et al., 2024, Xavier et al., 2022). For example, following changes in cannabis reform in Los Angeles and the District of Columbia, researchers observed a decrease in the absolute disparities for possession-related arrests, but an increase in the disparities of public-consumption arrests (Joshi et al., 2023). Further, PWUD report still experiencing volatile and unpredictable relationships with police, particularly unhoused PWUD (Good et al., 2025, Rouhani et al., 2026, Smiley-McDonald et al., 2024).

In Baltimore City, Maryland, where the current study is based, a prosecutorial *de facto* decriminalization policy implemented during the COVID-19 Emergency period was followed by substantial declines in misdemeanor arrests and charges (Rouhani et al., 2023). Though these declines were concentrated in the Black community, racial disparities widened among these declines, and qualitative research with people who use drugs (PWUD) found persisting fear and distrust of police (Rouhani et al., 2026). Particularly in the context of urban poverty, this emerging evidence suggests that the drugs themselves may not be the sole axis through which criminalization operates. Despite this, we have limited research that clarifies other mechanisms of criminalization for PWUD, such as survival-oriented legal informal and illicit income-generating activities to meet basic needs.

It is well documented that PWUD access multiple forms of broad ranging income generating activities, including ones that operate within criminalized economies (Abadie, 2026, Boyd et al., 2018, Dickson-Gómez et al., 2009; Richardson et al., 2015; Small et al., 2013). Despite this, the relationship between intersecting income strategies and harm-generating criminal legal exposure during drug decriminalization policy implementation, to the authors’ knowledge, remains underexplored and particularly for women. Women experience distinct forms of social and economic marginalization and often navigate diverse income networks, what this paper conceptualizes as *survival economic strategies*. These unique experiences may shape their exposure to the criminal legal system differently than men. For example, women may face heightened exposure to policing and criminalization due to greater engagement in child-related social assistance programs, where law enforcement often operates as a partner within the U.S. welfare system, as well as disproportionate involvement in illicit sex markets (Deering et al., 2011, Goldenberg, 2020, Huang et al., 2011, Sufrin, 2017). Examining the relationship between income sources and criminalization is then an important step toward filling in the empirical gap of mechanisms of criminalization for PWUD beyond the drug use itself, and towards illuminating the gendered vulnerabilities of women who use drugs (WWUD) to criminalization therein.

### 1.1 Survival economics: intersecting formal and informal economies

This study draws on the concept of survival economics, a term used to describe unsanctioned economic exchanges that develop on the margins of a formalized economy and are shaped by racial and socio-economic inequality (Grandpre, 2022, Hassan, 2022). Survival economies are typically described in urban contexts, such as predominantly Black neighborhoods in Chicago, where informal street-based (e.g., vending second-hand or hand-made items) and domestic (e.g., homemade food distribution) market participation has been defined to span both licit and illicit forms to meet neighborhood consumer demands for low-cost goods and services (Theodore, 2021, Venkatesh, 2006). The term conveys both deep scarcity that drives income generation, yet also reflects the nature of how individuals may draw from multiple economic spheres to leverage resources and survive broader economic inequality (Abadie, 2026).

Urban scholarship, largely qualitative in-depth research, has also shown that illicit economies, like illicit drug markets, are elaborately interconnected to non-illicit informal ones, like those that sell refurbished or handmade goods and govern panhandling and recycling, are also interconnected with formal institutions themselves (Venkatesh, 2006, Ziv, 2022). These scholars have documented that we cannot divorce informal economies from their formal counterparts, like the wage labor market, or forms of state assistance like food stamps and welfare that often intersect and shape the material conditions of deprivation, scarcity, and economic survival in the lives of the working and non-working poor. For example, low-income women often receive state assistance, have part-time formal employment, and participate in informal markets as laborers and consumers; therefore, generate income across multiple spheres. We refer as: survival economics (Edin and Lein, 1997, Knight, 2015).

### 1.2 Income generation, urban poverty, and the centrality of women of who use drugs

Population-based studies also indicate that PWUDs engage in multiple forms of income generation that span formal and informal economies (e.g., state assistance, drug selling), and with notable gender-based differences (DeBeck et al., 2007, Jaffe et al., 2021, Sherman and Latkin, 2002). For example, in a large urban cohort in Vancouver, Canada (n = 2231), Jaffe et al. (2021) found that men were significantly more likely to be engaged in street-based and illegal activities, whereas women more frequently relied on public assistance and sex work (Jaffe et al., 2021).

Despite extensive qualitative social scientific evidence on these overlapping economic spheres, few studies have examined how these intersecting income sources are combined and shape the experiences of criminalization among urban-dwelling women who use drugs. Existing epidemiological research has focused largely on Canadian urban settings, male-dominated populations, and isolated income types (Jaffe et al., 2021, Mitra et al., 2024). Attention to these relationships among women who use drugs (WWUD) is critical because unique income generating activities may differentially shape their pathways into criminalization and exposure to criminal legal harms differently than men (Haney, 2010, McKim, 2017). Gendered social roles, particularly caregiving responsibilities, can increase reliance on state assistance programs, such as cash welfare and food stamps while simultaneously exposing women to surveillance through child protective services during the course of drug use, creating distinct pathways into the criminal legal system (Huang et al., 2011, Sufrin, 2017). At the same time, economic precarity, drug dependence, and gendered power dynamics may increase reliance on informal sex exchange and other criminalized forms of income generation (Deering et al., 2011, Goldenberg, 2020). These gendered survival strategies are also associated with violence-and-drug related vulnerabilities, such as exposure to policing and arrest (Richardson et al., 2015). For example, women who sell drugs experience higher levels of violence than men, yet may be less likely to be detected by police or incarcerated (Ludwick et al., 2015, Small et al., 2013).

Characterizing how combinations of formal, informal, and illicit income sources intersect and examining if different types of income sources may be associated with criminal legal encounters, is a key step toward understanding the mechanisms that may limit drug and criminal legal policy reforms on WWUDs health and wellbeing, particularly in light of evidence illustrating persisting exposure to police under recent decriminalization policies (Good et al., 2025, Rouhani et al., 2026, Sisson et al., 2026, Smiley-McDonald et al., 2024).

### 1.3 Study purpose

The purpose of this study is to first, empirically characterize how four different income sources that span formal, informal, and illicit overlap, i.e., the *survival economics,* among WWUD in Baltimore City during the COVID-19 Federal emergency period and then to examine associations between independent income sources and criminal legal exposures during a period of *de facto* drug decriminalization.

## 2 Methods

### 2.1 Study population

Data for this analysis were drawn from the baseline interviews of the COVID Action Research Engagement (CARE) Study, which examined the longitudinal impacts of the COVID-19 pandemic among 227 WWUD in Baltimore, Maryland. Data collection occurred during the first two years of the COVID-19 federal emergency period (August 2021–December 2022), a time defined by overlapping public health and economic crises. In response to concerns about COVID-19 transmission in detention centers, Baltimore City implemented a non-prosecution policy that served as *de facto* decriminalization of several misdemeanor crimes (Rouhani et al., 2021, Rouhani et al., 2026). This policy temporarily halted prosecutions for several low-level, nonviolent offenses, including drug and paraphernalia possession, prostitution, and other poverty-related offenses (e.g., loitering, trespassing, public urination). As a result, study participants experienced the dual crises of the COVID-19 pandemic and the ongoing overdose epidemic, alongside the City’s decriminalization pilot.

Recruitment employed multiple strategies, including modified targeting sampling to identify locations with high levels of drug-related activity based on a secondary geospatial analysis of 911 calls related to drugs or drug use. Additional recruitment strategies included collaboration with service providers and other researchers, and windshield tours, during which study staff discreetly assessed potential recruitment zones for the visible presence of adult women. After identifying recruitment zones, trained study staff conducted field-based recruitment, consent, and enrollment in a mobile van. Interested individuals were screened in the study van to ensure privacy and confidentiality. Eligibility criteria included: (1) self-identifying as a woman; (2) 18 years or older; and (3) reporting non-medical use of opioids, crack cocaine, or powdered cocaine three or more times in the past three months. Eligible participants provided informed consent and completed a 45–60-minute interviewer-administered survey. Participants received a $50 Visa gift card as remuneration and, if desired, a bag containing harm reduction supplies and referrals to local resources. Comprehensive sampling procedures are described elsewhere (Tomko et al., 2022).

### 2.2 Measures

Measures ranging across a spectrum of survival economic strategies, in both formal and informal sectors, were guided by Edin and Lein’s, (1997) sociological framework. The framework identifies five primary financial resource categories common among low-income women to meet basic needs: state support, informal labor, formal employment, organizational assistance (e.g., non-profits, churches), and instrumental social support (e.g., family friends) (Edin and Lein, 1997).

#### 2.2.1 Latent class indicators of survival economics

Latent class indicators were drawn from a set of survey questions where participants were asked to select all primary income sources received over the past three months, including: public assistance (e.g., welfare, TCA, food stamps/SNAP, social security); part-time and full-time formal employment; informal employment (e.g., self-employment, off-the-books work, odd jobs); instrumental support (e.g., money from family, friends, romantic partners); child support; and illicit and acquisitive activities: sex work; panhandling; drug distribution (e.g., selling, touting, steering); and theft of goods (i.e., shoplifting, robbery, boosting).

Based on Edin and Lein’s framework, the final four indicators were categorized and defined as: state support (public assistance); informal labor market participation (odd jobs, self-employment, off-the-books work); instrumental support (financial assistance from family, friends, or romantic partners); and illicit income sources (sex work, panhandling, drug distribution, and theft). Overall, we captured three of their four framework categories and extended the model to include an additional income source specific WWUD (i.e., illicit income sources). Our dataset did not include organizational support and thus excludes that dimension. Following latent class analysis (LCA) conventions, income sources reported by fewer than ten percent of participants (i.e., child support, formal full time or part time employment) were also excluded. Given formal employment was reported by only seven percent and therefore omitted, informal work was retained for the analysis and relabeled under the labor market participation category as “informal labor market participation.”

#### 2.2.2 Covariates

Covariates of interest included socio-demographics (i.e., age, dichotomized at the mean of 40 years old), race and ethnicity (i.e., Non-Hispanic Black, Non-Hispanic White, or Hispanic) and no completion of a high school education (versus completion of high school education or greater). Structural vulnerability was defined as a reporting recent experience of homelessness, hunger within the last week, and having a disability that limited daily tasks. Pandemic-related challenges were reported from the onset of the emergency period if experienced household income loss, shifts in income sources, and temporary layoffs. Expanded safety net services during this period were elicited to include SNAP enrollment, receipt of a stimulus payment, and eligible-based unemployment benefits. Any alcohol use within the last month, currently smoking cigarettes, and recent illicit drug use (i.e., any drug injected, opioid, or fentanyl) were recounted. Recent recall was the past three months.

Last, our main covariates of interest are recent criminal legal exposures, reported as: (1) any arrest and (2) any police encounter. The latter was additionally examined as a subcategory: any negative police encounter. Exposure to policing encounters were collected using an index of police practices that was developed among a population of women who exchange sex in Baltimore City (Footer et al., 2020). These include any police practices (i.e., asked for ID, confiscated drugs, asked to move on, conducted search, and referred to health or social service) or any abusive practices (e.g., intimidation, destroyed property, touched inappropriately, pressured for sex to not arrest, accepted money, physically harmed/used weapon). Participants were asked whether they had encountered any of the discrete policing practices. Encounters are categorized and dichotomized into any (1 +) or no exposure. We additionally examined the binary sub-response to any police-mediated service referral to better understand the extent of a supportive encounter.

### 2.3 Analysis

First, we explored descriptive statistics (i.e., frequencies, proportions) across socio-demographic characteristics, primary income sources, experiences of structural vulnerability, pandemic-specific challenges, drug use, and criminal legal exposures (*n* = 227). Five women (2%) did not respond to income generation activities and therefore excluded from analyses.

Then, we characterized typologies of survival economics using LCA based on WWUD’ categorical responses (*n* = 222) to the four indicators capturing survival economic strategies: state support; informal labor market participation; social support; and illicit. This statistical approach identifies unobserved (latent) subgroups within a sample based on shared observed characteristics. In this case, it is the categorization of groupings of individuals based on similar forms of income generation strategies. To identify the most parsimonious set of distinct and interpretability groups, four class model solutions were fit and compared based on model fit criteria and interpretability. This was assessed according to a model’s Akaike Information Criteria (AIC), Bayesian Information Criteria (BIC), sample size-adjusted Bayesian Information Criteria (aBIC), Lo-Mendell-Rubin likelihood ratio test (LMR) and the bootstrap likelihood ratio test (BLRT). The smallest relative AIC, BIC and aBIC values exemplify better bit, including significant LMR and BLRT p-values, which compare the less parsimonious model to the larger one (e.g., three-class model vs. two). The entropy value provides information on the extent to which the identified classes distinguish and accurately classify a subject’s class membership. On a range of 0–1, values greater than 0.8 represent the highest classification accuracy and values ranging from 0.60 to 0.79 is acceptable if there is meaningful interpretation (Celeux and Soromenho, 1996). After the final model was selected, we estimated the probabilities of membership in each class as well as the probability of each indicator conditional on class membership. Five women were missing income values and dropped for the analysis.

Sensitivity analyses were conducted with formal employment and childcare. We ran two, three, and four class models to test whether these indicators may influence membership. There were no class changes, therefore retaining the original model for parsimony (results not presented).

Last, we explored covariates of interest associated with class membership using the manual three-step method accounting for Bolck, Croon, and Hagenaars (BCH) weights (e.g., experiences of homelessness, recent arrest) (Bakk and Kuha, 2021). This method allows for the correction of misclassification bias inherent in latent class membership. However, after running model associations with sociodemographic (e.g., homeless experiences) and recent arrests, we found that class solutions shifted and were not a reliable estimation (data not shown), possibly due to missing values and a small sample size. Therefore, we did not pursue further.

To further understand characteristics associated with different types of income generation, particularly with our outcomes of interest: criminal legal exposures, we explored associations with the two highest prevalent primary sources of income: illicit *vs* no illicit and state support *vs* no state support. For each of those income sources, we first explored descriptive covariate differences detected by significant *p-*values of chi-square tests of association at the *p* < 0.05 level. Then, we modeled the unadjusted and adjusted associations with criminal legal exposures (i.e., arrest, any police encounter, an abusive one). For both income source comparisons, we adjusted for age, race, education level, and recent injection drug use. The state support model included an additional adjustment for disability status. We also theorized that recent homelessness may confound the relationship for both income sources. However, we excluded it given that inclusion reduced the sample size, and similar effects were observed in parsimonious models. All models were run using Poisson regression with robust variance to calculate prevalence ratios because outcomes were greater than ten percent (Petersen and Deddens, 2008). Note that given the latent class findings of overlapping income, the exposed groups also include other income generating activities, for example, not just sole illicit income. We dropped one participant due to skipping an education question for a final sample of *n* = 221. These analyses were conducted in Stata 19.0 (StataCorp LLC, 2025) and the latent class analysis in Mplus version 8.1 (Muthén & Muthén., 2017).

## 3 Results

### 3.1 Sample characteristics

As seen in Table 1, participants (*n* = 227) were an average of 40 years old, reported being racially and ethnically diverse (46.7% Non-Hispanic (NH) Black, 37.0% NH white, and 16.3% Hispanic), and nearly half (40.1%) did not complete high school. Women were also highly structurally vulnerable with 30.4% reporting recently homelessness, 42.3% experiencing weekly hunger, and 34.4% reporting living with a disability. Because of the COVID-19 shutdown, 42.7% were laid off, 36.6% experienced a decrease in household income, 28.2% changed income sources, and 68.7% enrolled in SNAP. Half (49.8%) received a stimulus payment and 13.7% were eligible for and received unemployment. Self-reported opioid use was most prevalent (84.1%), followed by fentanyl use (57.3%). Eighty-five percent of women currently smoked, nearly one-fifth reported alcohol use in the last month (23.7%) and 34.8% reported injecting drugs. Criminal legal exposure was common: 17.6% reported an arrest; 43.6% a police encounter; and of those, 11.5% an abusive interaction, and 8.9% a police referral to health and social services.

**Table 1:** Descriptive statistics of 227 women who use drugs in Baltimore, MD during the COVID-19 Emergency Period and *de facto* drug decriminalization.

	*n (%)*
Total	*227*
**Socio-Demographics**	
Age, 40 years and older	108 (48.7)
*Race/ethnicity*	
Non-Hispanic Black	106 (46.7)
Non-Hispanic White	84 (37.0)
Hispanic	37 (16.3)
No completion of high school education^**^**^	93 (40.1)
**Structural Vulnerability during COVID-19**	
Homeless**	69 (30.4)
Laid off^**^**^	97 (42.7)
Experienced weekly hunger^**^**^	96 (42.3)
Household income decreased^**^**^	83 (36.6)
Disability status (yes)^**^**^	78 (34.4)
Changed income sources^**^**^	64 (28.2)
**Expanded Safety Net**	
SNAP enrollment^**^**^	156 (68.7)
Received stimulus payment^**^**^	113 (49.8)
Eligible for and received unemployment^**^**^	31 (13.7)
Substance Use*	
Opioid use	191 (84.1)
Fentanyl use	130 (57.3)
Injection drug use	79 (34.8)
Any alcohol use at least monthly	53 (23.7)
Current smoker	193 (85.0)
Criminal Legal Exposures*	
Any police encounter	99 (43.6)
Arrested	40 (17.6)
Any abusive police practice	26 (11.5)
Referral to health and social services	11 (8.9)
Primary income sources*^^^	
*Illicit sources*	
Selling sex	101 (44.5)
Panhandling	32 (14.1)
Selling, touting, or steering drugs	30 (13.2)
Boosting/shoplifting/robbery	26 (11.5)
*State Support*	118 (53.2)
*Instrumental support*	
Money from family or friends	91 (40.1)
Money from romantic partners	62 (27.3)
*Labor market participation*	
Informal employment (baby-sitting)	48 (21.6)
Part-time employment	12 (5.3)
Full-time employment	3 (1.3)
*Other*	
Child support	12 (5.3)

**missing greater than 10 (*n* = 13), proportions reflect missing values

^missing less than 5, proportions reflect missing values

⁎ recall past 3 months

The most reported primary source of income was state support (53.2%), followed by sex work (44.5%), instrumental support from family and friends (40.1%) and romantic partners (27.3%), and informal labor market participation (21.6%). Other sources of illicit income reported were panhandling (14.1%), drug distribution (13.2%), and boosting/shoplifting (11.5%). When creating Edin and Lein’s (1997) categories of income, the most reported was illicit forms (54.1%) followed by public assistance (53.2%), instrumental support (50.9%), and informal labor market participation (21.6%).

Experiencing *any type of police encounter* during the COVID-19 emergency and drug decriminalization period was the most prevalent reported criminal legal exposure (43.6%), following an arrest at 17.6%, and of those with a police encounter, 11.5% experienced an abusive encounter, and 8.9% were referred to health and social services by the police.

### 3.2 Characterizing survival economics

#### 3.2.1 Latent class solution

A three-class survival economic exploratory model was best identified based on substantive interpretability and fit statistics displayed in Table 2. First, based on the lowest BIC value (1149.951) and a high entropy value (0.803), a two-class model fit better than one, suggesting indicators of survival economics are best modeled as latent subgroups. In addition, statistically different LMR and BLRT tests provided evidence that the two-class solution fit better than one (respectively, *p* < 0.001 and *p* < 0.001) and a three-class fit better than two (respectively, *p* < 0.05 and *p* < 0.001). Then, compared to the second-class, the third-class had lower AIC (1115.221) and aBIC (1118.491) values. Given this information and that the third-class model provided greater conceptual differentiation among income types than the two-class solution, we selected a three-class solution. Also, the three- and four-class models had generally acceptable entropy values greater than 0.75. Moreover, the non-significant LMR and BLRT tests provided evidence that the four-class model was not statistically significant better fit compared to the more parsimonious three-class solution and it may be overparametrized (respectively, *p* = 0.1599 and *p* = 0.1031).

**Table 2:** Summary fit indices for one, two, three, and four latent class models of survival economics among women who use drugs in Baltimore, Maryland (n = 222) during the COVID-19 Emergency Period and *de facto* drug decriminalization.

Classes	s	Log Likelihood	AIC	BIC	aBIC	LMR p-value	BLRT p-value	Entropy	Smallest Class
1	4	-575.760	1159.519	1173.130	1160.453	n/a	n/a	n/a	n/a
2	9	-550.663	1119.327	**1149.951**	1121.429	0.0000	0.000	0.803	51.8%
3	14	-543.610	**1115.221**	1162.858	**1118.491**	0.0378	0.000	0.746	15.3%
4	19	-540.333	1118.666	1183.317	1123.104	0.1599	0.103	0.730	17.2%

Notes: Akaike Information Criteria (AIC), the Bayesian Information Criteria (BIC), sample size-adjusted Bayesian Information Criteria (aBIC), Lo-Mendell-Rubin likelihood ratio test (LMR), bootstrap likelihood ratio test (BLRT)

#### 3.2.2 Survival economic classes

Based on four indicators of singular income sources (state support, instrumental social support, informal market participation, and illicit income), three distinct survival economic classes were identified: (1) women who draw from *mixed and diverse income sources*, (2) those who access *primarily state support*, and (3) those whose income is derived *primarily from illicit sources*. Estimated class prevalences and conditional probabilities for each indicator are presented in Fig. 1 and Table 7. The first class, comprising 57% of the sample, are characterized by moderate to high probabilities across all income indicators, representing women who access multiple and diverse income streams. The second class (27%) is characterized by a high probability of receiving state support, representing women who primarily access formal assistance programs for financial support. The third class (15%) exhibits a high probability of earning income through illicit means and low probabilities across other sources, indicating a subgroup of women whose survival strategies rely primarily on illicit sources.

**Fig. 1:**
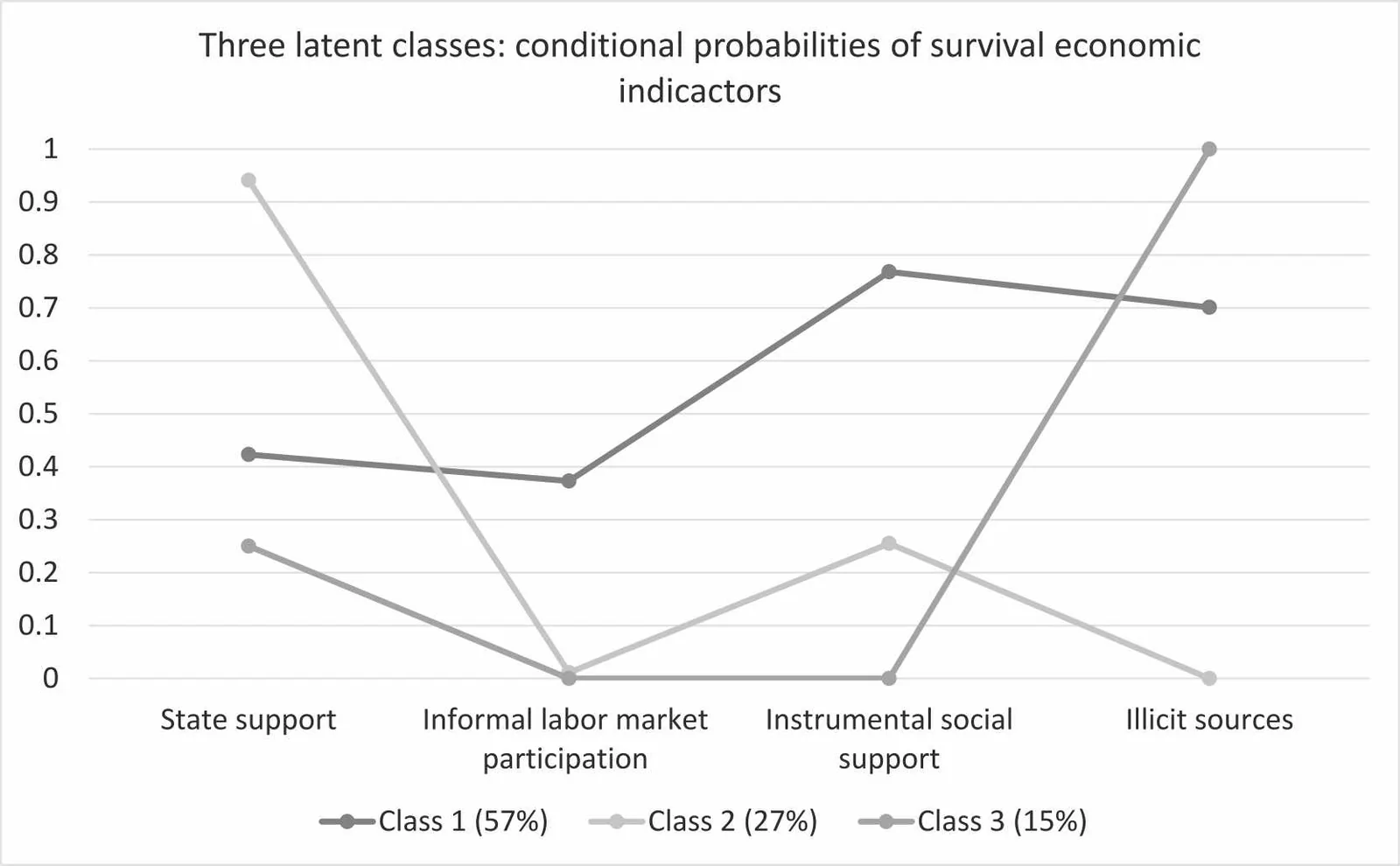
Three latent classes of survival economic strategies and conditional probabilities of income sources among women who use drugs in Baltimore City, Maryland during COVID-19 and defacto drug decriminalization (n = 222) Note: Class 1: Mixed and diverse income (57%), Class 2: Primarily state support (27%), Class 3: Primarily illicit sources (15%): Class prevalence (%), conditional probabilities (Y-axis)).

### 3.3 Correlates of two primary income sources: illicit and state support

*Illicit income versus no illicit income.* Descriptively, as reported in Table 3, women who reported illicit income were significantly more likely to be younger, Non-Hispanic White, structurally vulnerable (i.e., recently homeless and hungry), lost household income, was laid off and changed income sources during the pandemic, and reported recent injection drug use, compared to women who did not report illicit sources, all respectively *p* < 0.05. *State support versus no state support.* As seen in Table 4, Women who reported state support were significantly more likely to be older, report having a disability, enroll in SNAP during the pandemic, report recent injection drug use, and less likely to be homeless, relative to women who did not report this income source, all respectively *p* < 0.05. Alcohol use in the past month was also marginally significant and more common among women who reported state support versus those who did not (*p* = 0.056)

**Table 3:** Descriptive statistics of 222 women who use drugs in Baltimore, MD by illicit income.

	*Illicit income*	*No illicit income*	
	*n (%)*	*n (%)*	*p-value*
Total	*123*	*99*	
**Socio-Demographics**			
Age, 40 years and older	**43 (34.9)**	**65 (65.7)**	**< 0.001**
*Race/ethnicity*			
Non-Hispanic Black	**45 (36.6)**	**59 (59.6)**	
Non-Hispanic White	**60 (48.8)**	**23 (23.2)**	
Hispanic	**18 (14.6)**	**17 (17.2)**	**< 0.001**
No completion of high school education^**^**^	56 (45.5)	35 (35.4)	0.141
**Structural Vulnerability during COVID-19**			
Homeless**	**52 (43.7)**	**15 (16.3)**	**< 0.001**
Experienced weekly hunger^**^**^	**66 (54.6)**	**30 (30.3)**	**< 0.001**
Household income decreased^**^**^	**54 (44.3)**	**28 (28.3)**	**0.014**
Changed income sources^**^**^	**46 (37.7)**	**16 (16.3)**	**< 0.001**
Laid off^**^**^	**59 (48.0)**	**34 (34.7)**	**0.047**
Disability status (yes)^**^**^	37 (30.6)	41 (41.8)	0.084
**Expanded Safety Net**			
SNAP enrollment^**^**^	81 (67.5)	73 (73.7)	0.315
Received stimulus payment^**^**^	56 (46.7)	54 (54.6)	0.246
Eligible for and received unemployment^**^**^	21 (17.5)	10 (10.2)	0.125
Substance Use*			
Opioid use	109 (92.4)	79 (86.8)	0.185
Fentanyl use	77 (71.9)	51 (61.5)	0.125
Injection drug use	**56 (45.5)**	**22 (22.2)**	**< 0.001**
Any alcohol use at least monthly	26 (21.5)	27 (27.3)	0.318
Current smoker	105 (86.8)	84 (84.9)	0.683

Note: Given the latent class findings of overlapping income, illicit income also includes other income generating activities, not just sole illicit income.

*recall past 3 months

Boldface: statistically significant, statistical associations with chi-square tests, *p* < 0.05

**missing greater than 10 (*n* = 13)

^missing less than 5, proportions reflect missing values

**Table 4:** Descriptive statistics of 222 women who use drugs in Baltimore, Maryland by state support.

	*State support*	*No state support*	
	*n (%)*	*n (%)*	*p-value*
Total	*118*	*104*	
**Socio-Demographics**			
Age, 40 years and older	**68 (57.6)**	**40 (38.5)**	**0.004**
*Race/ethnicity*			
Non-Hispanic Black	62 (52.5)	42 (40.4)	
Non-Hispanic White	42 (35.6)	41 (39.4)	
Hispanic	14 (11.9)	21 (20.2)	0.111
No completion of high school education^**^**^	47 (40.2)	44 (42.3)	0.747
**Structural Vulnerability during COVID-19**			
Homeless^**^**^	**28 (25.2)**	**39 (39.0)**	**0.035**
Experienced weekly hunger^**^**^	45 (38.8)	51 (49.0)	0.126
Household income decreased^**^**^	45 (38.5)	37 (35.6)	0.658
Changed income sources^**^**^	33 (28.2)	29 (28.2)	0.993
Laid off^**^**^	47 (40.2)	46 (44.2)	0.542
Disability status (yes)^**^**^	**49 (42.6)**	**29 (27.9)**	**0.023**
**Expanded Safety Net**			
SNAP enrollment^**^**^	**97 (83.6)**	**57 (55.3)**	**< 0.001**
Received stimulus payment^**^**^	65 (56.0)	45 (43.7)	0.068
Eligible for and received unemployment^**^**^	20 (17.2)	11 (10.8)	0.173
Substance Use*			
Opioid use	98 (86.1)	90 (90.9)	0.662
Fentanyl use	66 (65.4)	62 (69.7)	0.527
Injection drug use	**45 (43.3)**	**33 (28.0)**	**0.017**
Any alcohol use at least monthly	34 (29.3)	19 (18.3)	0.056
Current smoker	99 (85.3)	90 (86.5)	0.799

Note: Given the latent class findings of overlapping income, state support also includes other income generating activities, not just sole state support.

*recall past 3 months

Boldface: statistically significant, statistical associations with chi-square tests, *p* < 0.05

**missing greater than 10

^missing less than 5, proportions reflect missing values

### 3.4 Adjusted associations with criminal legal exposures

*Illicit income versus no illicit income*. In the adjusted models, WWUD who reported illicit income sources (Table 5), in contrast to those who did not, were statistically significantly more likely to report any police or abusive police encounter (borderline significant) after adjusting for age, race, education level and recent injection drug use (respectively adjusted prevalence ratios (aPR) 1.61 95% Confidence Interval (CI) 1.39, 2.01, *p* < 0.001 and aPR 1.33 95% CI 1.02,1.74, *p* < 0.05). There were no adjusted differences for arrests (aPR 1.15 95% CI 0.92, 1.45, *p* = 0.228). *State support versus no state support.* On the other hand, as shown in Table 6, there were no statistically significant differences in criminal legal exposures among women who accessed state support, versus those who did not, in the adjusted models.

**Table 5:** Unadjusted and adjusted associations between illicit income and criminal legal exposures among women who use drugs (*n* = 221) in Baltimore, Maryland during the COVID-19 emergency period and *de facto* drug decriminalization.

		Illicit income vs. no illicit income			
		*Unadjusted*		*Adjusted*	
	*n* (%)	Prevalence Ratio (95% CI)	*p*	Prevalence Ratio (95% CI)	*p*
Arrested	38 (17.2)	**1.30 (1.00, 1.68)**	**0.046**	1.15 (0.92, 1.45)	0.228
Any police encounter	97 (43.9)	**1.65 (1.30, 2.09)**	**< 0.001**	**1.61 (1.29, 2.01)**	**< 0.001**
Any abusive police practice	26 (11.8)	**1.46 (1.14, 1.88)**	**0.003**	**1.33 (1.02, 1.74)**	**0.031**

Note: adjusted for age, race, education level (missing=1), and recent injection drug use. Additional models were run to include recent experiences of homelessness as a confounder, but due to a reduced sample size and similar effects, we report the parsimonious adjusted model. Boldface: statistically significant, statistical associations at *p* < 0.05

**Table 6:** Unadjusted and adjusted associations between state support and criminal legal exposures among women who use drugs (*n* = 221) in Baltimore, Maryland during the COVID-19 emergency period and *de facto* drug decriminalization.

		State support vs. no state support			
		*Unadjusted*		*Adjusted*	
	*n* (%)	Prevalence Ratio (95% CI)	*p*	Prevalence Ratio (95% CI)	*p*
Arrested	38 (17.2)	1.17 (0.88, 1.57)	0.288	1.25 (0.92, 1.63)	0.157
Any police encounter	97 (43.9)	**0.77 (0.59, 0.99)**	**0.047**	0.79 (0.61, 1.04)	0.096
Any abusive police practice	26 (11.8)	0.69 (0.422, 1.15)	0.162	0.72, (0.42, 1.25)	0.245

Note: adjusted for age, race, education level (missing=1), recent injection drug use, and disability status. Additional models were run to include recent experiences of homelessness as a confounder, but due to a reduced sample size and similar effects, we report the parsimonious adjusted model. Boldface: statistically significant, statistical associations at *p* < 0.05

**Table 7:** Class-specific conditional probabilities and standard errors (SE) for the 3-class LCA model of survival economics among women who use drugs in Baltimore City, MD during COVID-19 and *de facto* drug decriminalization (*n* = 222).

Income indicator	Class 1 Mixed and diverse income (57%, n = 126)	Class 2 Primarily state support (27%, n = 61)	Class 3 Primarily illicit sources (15%, n = 34)
State support	0.423 (SE 0.05)	0.941 (0.09)	0.250 (0.09)
Labor market participation (informal)	0.373 (0.06)	0.011 (0.04)	0.000 (0.00)
Instrumental social support	0.768 (0.07)	0.255 (0.09)	0.000 (0.00)
Illicit sources	0.701 (0.07)	0.000 (0.00)	1.000 (0.00)

## 4 Discussion

This exploratory study is the first to characterize the survival economic strategies of women who use drugs (WWUD) using a latent class approach and to explore the relationship between income sources and criminal legal encounters. Findings identified three distinct income classes during this time period, illustrating the intersections of women’s economic survival strategies and reflecting the well-documented qualitative tradition that describe the interdependence of formal, informal, and illicit income opportunities that WWUD often navigate to secure material needs in urban cities and may place them under unique criminal legal circumstances (Ludwick et al., 2015, Venkatesh, 2006, Ziv, 2022). By empirically modeling these patterns, we build on studies that often treat employment, state support, and illicit income as discrete domains (DeBeck et al., 2007, Jaffe et al., 2021, Small et al., 2013) and offer foundational and exploratory evidence that WWUD in Baltimore simultaneously generate income in intersecting ways which may subject them to criminalization even amid many contemporary reforms. These findings suggest a multifaceted, activity-based (ather than person-based) understanding of the criminalization of PWUD, with important implications for future research and policy.

This study extends previous ethnographic research on the overlapping nature of income generation that occurs in “inner-city drug scenes” among WWUD in Baltimore and adds to a growing literature base about factors which may affect drug decriminalization reforms without structural investments and redistributions of power and resources (Fast et al., 2017, Flath et al., 2025, Michaud et al., 2024, Rouhani et al., 2026, Ziv et al., 2023). Despite living through a *de facto* decriminalization period where police did not have a prosecutorial route to criminalize drugs or other crimes of poverty, police interactions among women were still much higher than arrests, suggesting continued exposure to policing, and they were statistically significantly higher for those engaging in illicit economies, such as sex work, whether abusive or not. This is similar to previous research that has documented exorbitantly high rates of incarceration among sex workers in Baltimore, who are more likely to experience police violence (Fehrenbacher et al., 2020). Although women who accessed state support were not more likely to experience police interactions compared to those who did not, there was overlap between groups as shown in the latent class results, underscoring the interconnectedness between income generation and policing among WWUD and the novelty of extending this literature by documenting police experiences across diverse income generation. The finding that women generate income through numerous methods illustrates that several other mechanisms may exist, beyond drugs, which can lead to continued criminalization of WWUD even amid drug decriminalization efforts.

The high prevalence of police encounters during this period is consistent with emerging evidence that policing of people who use drugs often persists despite drug decriminalization. Studies from British Columbia, Sydney, and Oregon have documented continued, persistent police surveillance, civil enforcement, monitoring of street-based survival activities (e.g., homelessness) and, at times, retaliatory policing despite decriminalization (Good et al., 2025, Pamplin et al., 2023, Xavier et al., 2022). Rather than eliminating criminal legal contact, these studies have observed that decriminalization often shifts policing away from drug possession toward other criminalized survival activities, such as selling loose cigarettes or other street-based informal activities (Good et al., 2025, Speed et al., 2025). Similarly, a recent qualitative study among people who use drugs in Baltimore found that although participants perceived a reduced risk of arrest for drug possession, the nature of street-level interactions with police remained highly unpredictable and concerns about police surveillance, harassment, and misconduct remained widespread following *de facto* decriminalization (Rouhani et al., 2026). Our exploratory and associative study extends this work by highlighting a unique mechanism that may explain the continued exposure to policing, via survival economics, and warrants further confirmation in future research and policy making.

Another potential mechanism is police discretion, through which racial disparities in policing may persist following drug decriminalization, as documented in a recent scoping review of more than sixty studies (Jurkus et al., 2026). In our study, White WWUD were more likely than non-White WWUD to report illicit income (compared to those who did not), and those reporting illicit income were more likely to encounter police. Although this pattern contrasts with national evidence of disproportionate drug-related arrests among Black people who use drugs despite similar levels of drug use (Rouhani et al., 2024), it highlights the importance of considering how specific income-generating activities may shape police exposure and their intersections with race, class, and gender. Compared to women who did not receive state support, those who did were no more likely to encounter police, and there were no racial/ethnic differences in these associations, suggesting that street-based illicit activities, such as sex work and panhandling, may be particularly important sources of police exposure, warranting further research on this.

Our findings also contributes to nascent literature examining the gendered social and economic conditions shaping WWUD’s survival economies and overdose vulnerability (Goldenberg, 2020, Terplan, 2017). Using repeated-measures latent class analysis, Mitra et al. (2024) identified distinct patterns of marginalization among women who use drugs in Vancouver, finding that women characterized by high illicit income generation and criminal legal involvement experienced the greatest risk of non-fatal overdose, whereas those with stable employment and limited street-based work had the lowest risk (Mitra et al., 2024). Although we did not examine overdose outcomes, we similarly found that women engaged in illicit income generation were more likely to report police encounters, while women relying on state assistance generally did not experience elevated police exposure. Despite differences in study design, both studies are exploratory latent models and demonstrate that women may navigate diverse survival economic strategies with important implications for criminal legal exposure and health, underscoring the need for confirmatory analyses across different contexts to inform innovative, place-based public health and drug policy interventions.

### 4.1 Limitations and opportunities for future research

This study has several limitations. First, the modest sample size and missing data precluded examination of covariates associated with latent class membership. Consequently, we were unable to assess whether women engaging in more complex survival economic strategies (e.g., drawing on multiple income sources) experienced greater criminal legal exposure than those relying primarily on state support. To address this limitation, we examined associations between two prevalent income sources that touch on both points of the spectrum of formal and illicit activities: state support and illicit income, that broadly represented the identified survival strategies. Although these measures should not be interpreted as substitutes for the latent classes, they provide insight into potential mechanisms linking income generation and criminalization. Future studies with larger samples are needed to validate these survival economic typologies and examine predictors and consequences of class membership.

Second, findings should be interpreted within the unique context of the COVID-19 pandemic and may not generalize to post-pandemic conditions. COVID-19 intensified existing economic hardship among women who use drugs through service disruptions, labor market instability, social isolation, and an increasingly toxic drug supply, while temporary expansions in federal assistance improved access to a safety net (Clouston et al., 2021, Connor et al., 2020, Devoto et al., 2022). Consistent with these broader trends, nearly half of participants reported job loss and almost two-thirds received SNAP benefits. Formal employment was rare in this sample and was therefore excluded from the final survival economies framework, limiting its ability to capture the full range of income-generating strategies typically available to low-income women. Although our findings are consistent with pre-pandemic studies documenting limited formal employment and substantial reliance on public assistance among people who use drugs (Richardson et al., 2010), additional research is needed to determine whether the observed survival economic patterns and their associations with criminal legal exposure persist beyond the pandemic. For example, while we observed differences in police exposure across income sources, it remains unclear whether these patterns reflect pandemic-related disruptions, Baltimore’s *de facto* decriminalization policies, or more persistent structural dynamics. Additionally, data were collected between August 2021 and December 2022, a period of substantial change in employment opportunities and economic conditions associated with the COVID-19 pandemic. Thus, findings should be interpreted within the context of this changing pandemic-era environment and again, may not reflect conditions outside this period.

Third, the observed associations are exploratory and should be interpreted with caution. The cross-sectional design precludes establishing the temporal ordering between criminal-legal exposure and income generation. Although measures reflected experiences within a relatively short three-month recall period and provides some temporal proximity between them, we cannot determine whether criminal-legal contact preceded or followed reported income-generating activities. Mixed-methods longitudinal research is needed to clarify these potential bidirectional relationships.

Fourth, the absence of a pre-policy or comparison group and the lack of information on the reasons for police contact or arrest preclude determining whether observed criminal-legal exposures were attributable to, or persisted despite, drug decriminalization. Moreover, because prosecutorial non-prosecution policies do not necessarily prevent police encounters or arrests, our findings should be interpreted as describing criminal-legal exposure during the policy period rather than as evidence of the effects of decriminalization. Future research should clarify how policing persists during decriminalization, including through the policing of criminalized survival activities.

Fifth, the small number of participants reporting abusive policing (n = 26) may limit the precision and stability of the adjusted model estimates; therefore, those associations should be interpreted as exploratory and with particular attention to the borderline confidence interval at 1.02. The observed associations between illicit income and policing, including both any and (marginally significant) abusive policing encounters, underscore the importance of examining the breadth and range of policing practices affecting people who use drugs and their broader communities in future research.

Finally, given this was an exploratory analysis conducted during the COVID-19 pandemic and our data did not capture the full spectrum of income-generating strategies in Edin’s framework, future confirmatory research should use more comprehensive measures and non-pandemic samples to assess the stability and generalizability of these classes while minimizing potential overparameterization.

Despite these limitations, the study captures a hard-to-reach population during an unprecedented period of social and economic disruption, providing an important baseline for understanding survival economies among women who use drugs and for understanding the long-term reverberations of large-scale global disruptions on the health and wellbeing of WWUD (Richardson et al., 2016). While emergency conditions have subsided, public health scholars argue that structural inequities exposed during that time persist and we must continue to anticipate and learn from them (Friedman et al., 2021). Therefore, the findings establish an empirical foundation for future research to examine how women's income-generating strategies evolve over time and how they shape exposure to criminal legal surveillance across changing social and policy contexts.

That said, replication in these and other similarly situated settings is needed to assess the generalizability and stability of the patterns and associations identified here. Given the exploratory nature of this study and its non-probability sampling approach, in which women were recruited from locations identified through drug-related 911 calls, the findings should not be broadly generalized to all women who use drugs in Baltimore or elsewhere. This recruitment strategy may have preferentially captured women living in or frequenting neighborhoods with higher levels of drug activity and concentrated law enforcement. Thus, replication is needed and may be particularly relevant and important to conduct in other post-industrial urban settings characterized by similar histories of racialized disinvestment, concentrated poverty, open-air drug markets, and targeted law enforcement in marginalized neighborhoods, such as Milwaukee, Philadelphia and Chicago.

### 4.2 Policy recommendations

Our findings, while exploratory, contribute to growing evidence that centering the criminalization of drugs themselves through use and possession alone *may* not address the broader structural and economic conditions shaping criminal legal exposure and their subsequent harms (Flath et al., 2025, Good et al., 2025, International Network of People who use Drugs, 2021, Michaud et al., 2024). Prior critiques similarly suggest that drug policy reforms, such as Good Samaritan Laws, may be insufficient to address structural racism and intersecting mechanisms of criminalization affecting racially minoritized communities (Pamplin et al., 2023). In the present study conducted during a period of *de facto* drug decriminalization reform in Baltimore when drug possession arrests declined citywide (Rouhani et al., 2023), women who relied on criminalized survival economies still reported encounters with police. Additionally, a separate analysis using this sample found that increasing overlapping structural vulnerabilities and material hardship during COVID-19 were associated with significantly greater likelihood of experiencing multiple forms of policing and enforcement (Sisson et al., 2026). The likelihood of experiencing two or more enforcement practices was 5.5 times greater among women who experienced both challenges accessing harm reduction supplies and lacking a bathroom, increasing to 7.1 times greater among those who additionally experienced challenges finding a place to stay.

Although our cross-sectional design does not permit causal or pre-post assessment of the policy’s effects, these findings highlight the need for further research that clarifies how policing may persist during decriminalization, including through the policing of criminalized survival activities. They also align with broader calls to pair drug decriminalization with social and economic policies that expand access to stable livelihoods, strengthen collective mobilization among WWUD, support autonomous care-based economic systems, and reduce the criminalization of gender-based labor (i.e., sex work and other street-based activities) (Austin et al., 2023, Brown and Riley, 2005, Goldenberg, 2020, Hassan, 2022).

Our findings also highlight the potential value of evaluating emerging non-police response models and other community-led approaches alongside decriminalization reforms to reduce reliance on police and their associated harms, including community-led overdose and emergency medical response (Boland et al., 2023, Heidari et al., 2026, Oakland Power Projects, 2025). While these approaches are rapidly proliferating (Balfour et al., 2022), it remains unclear to what extend they represent extensions of police power rather than true alternatives (Erikson et al., 2025). Future research should therefore examine whether and how non-police and community-led approaches affect criminal legal exposure among WWUD while attending to the potential for net-widening and unintended forms of surveillance that can create new barriers to income generation, safety and survival.

As we continue to innovate, it may be worthwhile to develop decriminalization strategies to accompany reparative investments and address the socio-economic consequences of the drug war (Flanigan and Freiman, 2023, King and Page, 2018). Community-led reinvestment, or so called *reparations for the war on drugs*, in housing, employment, harm reduction, and culturally grounded systems of care are currently in pilot stages and may help alleviate the structural conditions that shape both economic marginalization and criminal legal exposure (Flath et al., 2025, Hawks et al., 2022).

## 5 Conclusions

This exploratory study identified distinct survival economic strategies among women who use drugs and observed notable associations between illicit income generation and criminal legal exposure during a period of drug decriminalization reform in Baltimore City amid the COVID-19 pandemic. Women engaged in illicit income-generating activities were more likely to report police encounters, whereas accessing state support was not associated with increased police exposure. Although our design cannot determine whether these patterns were attributable to or persisted because of decriminalization, our findings align with broader evidence outlining that criminal-legal exposure may occur through mechanisms beyond drug possession enforcement alone. These exploratory findings underscore the need to confirm how criminalized survival economies are policed. Most importantly, they underscore the need to consider strategies that reduce criminal-legal harms broadly and advance health equity among women who use drugs.

## CRediT authorship contribution statement

**Catherine Tomko:** Writing – review & editing, Writing – original draft, Supervision, Project administration, Data curation. **Tali Ziv:** Writing – review & editing, Writing – original draft. **Natalie Flath:** Writing – review & editing, Writing – original draft, Visualization, Methodology, Formal analysis, Conceptualization. **Sherman Susan:** Writing – review & editing, Writing – original draft, Supervision, Investigation, Funding acquisition, Data curation. **Sisson Laura:** Writing – review & editing, Writing – original draft, Methodology. **Saba Rouhani:** Writing – review & editing, Writing – original draft.

## Ethics declaration

Written informed consent to take part in the study and to publish the article has been obtained from all participants or their legal representatives. The privacy rights of participants have been observed.

This study was performed in compliance with relevant laws, regulatory frameworks and guidelines where the research took place. This study was approved by the Johns Hopkins Bloomberg School of Public Health Institutional Review Board. (Approval No. IORG 0000077 and FWA 00000287)

## Ethics approval

All study activities, including COVID-19 safety protocols for data collection, were approved by the Johns Hopkins Bloomberg School of Public Health Institutional Review Board.

## Declaration of Competing Interest

The authors declare the following financial interests/personal relationships which may be considered as potential competing interests: Susan G. Sherman serves as an expert witness in opioid litigation. No other authors have any disclosures, competing financial interests or personal relationships that influence the work reported in this paper.

## Data Availability

To protect participant privacy, data for this study is not publicly available due to the sensitive nature of information gathered.
